# Chronic Variable Stress Is Responsible for Lipid and DNA Oxidative Disorders and Activation of Oxidative Stress Response Genes in the Brain of Rats

**DOI:** 10.1155/2017/7313090

**Published:** 2017-09-11

**Authors:** Mariola Herbet, Agnieszka Korga, Monika Gawrońska-Grzywacz, Magdalena Izdebska, Iwona Piątkowska-Chmiel, Ewa Poleszak, Andrzej Wróbel, Włodzimierz Matysiak, Barbara Jodłowska-Jędrych, Jarosław Dudka

**Affiliations:** ^1^Chair and Department of Toxicology, Medical University of Lublin, Chodźki 8, 20-093 Lublin, Poland; ^2^Department of Applied Pharmacy, Medical University of Lublin, Chodźki 1, 20-093 Lublin, Poland; ^3^Second Department of Gynecology, Medical University of Lublin, Jaczewskiego 8, 20-090 Lublin, Poland; ^4^Department of Histology and Embryology, Medical University of Lublin, Radziwiłłowska 11, 20-080 Lublin, Poland

## Abstract

Chronic environmental stress is associated with reactive oxygen species (ROS) overproduction and the pathogenesis of depression. The purpose of this study was to evaluate biochemical and molecular changes associated with ROS generation in the brains of rats submitted to chronic variable stress. Male Wistar rats (50–55 days old, weighing 200–250 g) were divided in two groups (*n* = 10): control and stressed. Rats in the stressed group were exposed to stress conditions for 40 days. The animals were decapitated and the brain samples were collected. In prefrontal cortex, we measured the following biochemical parameters: lipid peroxidation and concentration of glutathione—GSH, GSSG, GSH/GSSG ratio, glutathione peroxidase, and glutathione reductase activities. In the hippocampus marker of DNA, oxidative damage and expression of DNA-repairing genes (*Ogg1*, *MsrA*) and gene-encoding antioxidative transcriptional factor (*Nrf2*) were determined. The results demonstrate indirect evidence of ROS overproduction and presence of oxidative stress. They also reveal disruption of oxidative defense systems (decreased GR activity, diminished GSH/GSSG ratio, and decreased *Nrf2* expression) and activation of the oxidative DNA repair system (increased *Ogg1* and *MsrA* expression). Together, the presented data suggest that independent activation of oxidative stress response genes occurs in chronic variable stress conditions.

## 1. Introduction

Depression is currently the most common affective disorder. It is estimated to affect over 120 million people worldwide and the number of cases is steadily increasing [1]. Forecasts indicate that by 2020, it will rank second in lifestyle diseases that reduce the capacity to work [2]. Known causes of depression do not provide sufficient explanation of pathophysiology, despite extensive research in this area. It is believed that the process is multifactorial and has many subtypes with more than one etiology. Studies have shown that chronic stress is directly implicated in the pathogenesis of depression [3, 4]. Stressful events may induce multiple behavioral, neurochemical, and biological alterations, presumably as an adaptive response to meet environmental demands. It has been described that a prolonged and sustained stimulation, caused by stress exceeding the body's capacity to maintain homeostasis, can result in psychopathological events [5].

It is postulated that chronic environmental stressors may have a significant impact on reactive oxygen species (ROS) generation in the brain [6, 7]. Studies have consistently reported an increase of ROS in the blood of patients with major depression [8]. The mechanism underlying the ROS increase remains unclear and is likely mediated in part by stress-related hormones such as cortisol [9]. A stress-induced increase in cortisol levels has been reported to accelerate glucose metabolism and the production of ROS [10]. ROS, such as superoxide anion (O_2_^−^), hydrogen peroxide (H_2_O_2_), and hydroxyl radicals (HO•), are extremely reactive, due to unpaired electrons. Hydrogen peroxide is particularly damaging the DNA as it is less reactive than other radicals and able to travel into the cell nucleus, subsequently reacting with macromolecules such as DNA. In well-functioning central nervous system (CNS) cells, there is a balance between the formation of free radicals and their scavenging. The shift in this equilibrium toward the formation of ROS generates oxidative stress, which is defined as an increase in oxidation potential to the level leading to macromolecular oxidation (e.g., lipids, DNA, and protein), despite adaptive activation of antioxidative defense system [11, 12]. Oxidative stress leads to the formation of lipid peroxidation products, resulting in the loss of cell membrane fluidity, reduced membrane potential and possible rupture. Upon rupture, cell and organelle contents are released into the extracellular space, including neurotransmitters (serotonin and noradrenaline) related to major depression [13]. Subjecting cells to oxidative stress can result in cell membrane modifications and leads to disruption of receptor functions and enzyme and gene activity [14]. Oxidation-modified compounds and molecules that disturb cellular homeostasis can lead to apoptosis or necrosis [15, 16]. These events are related to various neurodegenerative diseases [3, 14, 17]. Neuronal cells in the brain are highly sensitive to oxidative stress due to a large dependence on oxidative phosphorylation for energy compared to other cell types. Normal brain function is dependent on a continuous and efficient use of oxygen. Although the brain represents only 2% of human body weight, it consumes 20% of the total body oxygen with 1-2% of the oxygen being converted into superoxide anion radicals and hydrogen peroxide. Increased O_2_^∗−^ production in the mitochondria inhibits the Krebs cycle through temporary aconitase inactivation. The Krebs cycle is inhibited by lipid peroxidation products—MDA and 4HNE [18, 19]. Moreover, MDA and 4HNE can cause DNA damage [20]. It is worth mentioning that the brain as an organ is a major metabolizer of oxygen (20% of the body consumption) and yet has relatively feeble protective antioxidant mechanisms [21].

One of the most important consequences of ROS overproduction and chronic oxidative damage is DNA modification, which can become permanent via the formation of mutations and other types of genomic instabilities [22–25]. Studies have shown that modulation of gene expression under oxidative stress conditions is an important mechanism in depression [24–27]. Among various types of DNA base modification induced by ROS, 7, 8-dihydro-8-oxoguanine (8-oxoG) is the most widely studied and is considered to be a key biomarker of oxidative DNA damage [14, 27, 28]. Studies have revealed significantly elevated levels of 8-OxoG in urine of depressed patients in comparison to healthy controls [29]. The 8-oxoguanine glycosylase1 (OGG1) gene is a key component of the base excision repair pathway, because OGG1 encodes the enzyme responsible for the excision of 8-oxoguanine, a mutagenic base byproduct that occurs as a result of exposure to reactive oxygen [22]. Methionine sulfoxide reductase A (MSRA) also plays an important role in the repair of oxidatively damaged proteins toward restoration of biological activity [23]. MSRA reduces methionine sulfoxide (MetO) to methionine. Because methionine residues are particularly susceptible to oxidation by ROS, MSRA has important functions in cellular metabolism: as an antioxidant enzyme that scavenges ROS by facilitating the cyclic interconversion of methionine between oxidized and reduced forms and as a repair enzyme by keeping critical methionine residues in their reduced form [30, 31]. Several studies have demonstrated high levels of *MsrA* expression in brain neurons and confirmed that *MsrA* overexpression results in an extension of lifespan in mice and in human T cells under conditions of oxidative stress [23, 31]. An emerging regulator of cellular resistance to oxidants is nuclear factor erythroid 2-related factor 2 (NRF2). NRF2, beyond its regulatory role in antioxidant enzyme expression, has been recognized as a key factor in the regulation of an array of genes that defend cells against the deleterious effects of environmental insults [32]. An important mechanism of cellular defense against oxidative or electrophilic stress is achieved through activation of the Nrf2-antioxidant response element signaling pathway. The pathway controls the expression of genes whose protein products are involved in the detoxification and elimination of reactive oxidants through enhancing cellular antioxidant capacity [33–35]. By regulating oxidant levels and oxidant signaling, NRF2 participates in the control of mitochondrial biogenesis [36]. Moreover, NRF2 likely also affects mitochondrial ROS production [37]. Increased expression of *Nrf2* is an important preventative component in neurodegenerative diseases [38]. Lowering NRF2 activation may reduce antioxidative responses [39].

The role of oxidative stress in depression appears to be associated with neurogenesis and cell survival, but whether oxidative stress is a cause or merely a downstream consequence of the neurodegenerative process still remains unexplained [23]. It has been hypothesized that chronic environmental stress-induced oxidative stress may contribute to changes at the molecular level. The molecular studies seem to be crucial in this regard. Chronic oxidative damage can be indirectly assessed by measuring changes in the expression of genes involved in protection and repair systems.

In consideration of these findings, the main objective of our study was the assessment of oxidative DNA damage in the brain of rats submitted to chronic variable stress. The extent of AP sites (one of the major types of damage generated by ROS) and *Ogg1*, *MsrA*, and *Nrf2* genes was measured. In the present study, we also investigated the effect of chronic environmental stress on lipid peroxidation, glutathione redox status, and antioxidant enzyme activities (glutathione peroxidase and reductase). We used the prefrontal cortex to determine biochemical parameters and hippocampus for molecular tests because depressed patients present alterations in these cerebral structures and relevant research most often relates to these regions of the brain [40–42]. The chronic variable stress (CVS) paradigm is a well-validated animal model of depression, as recent publications have confirmed that CVS can induce behavioral and neurochemical changes in animals that are similar to the symptoms and presumed neurochemical changes accompanying depression in humans [43–45]. Similarly, clinical evidence describes that stressful life events, which significantly increase the risk of depressive episodes, are generally of a chronic nature [46, 47].

## 2. Materials and Methods

### 2.1. Animals

Male Wistar rats, weighing 200–250 g, approximately 50–55 days old at the time of arrival, purchased from a licensed breeder (Brwinów, Poland) were used as test subjects. The animals were housed in standard rectangular polypropylene cages with standard diet and water available ad libitum. The colony room was maintained at a constant temperature (20 ± 2°C) under a 12 h day/12 h night cycle in constant environmental conditions (humidity, noise). All experimental procedures were approved by the Local Ethics Committee on Animal Experimentation of the Medical University of Lublin (number 12/2015) and were performed in accordance with obligatory European standards related to the experimental studies on animal models.

### 2.2. Chronic Variable Stress Procedure

A chronic variable stress protocol was carried out as described by Gamaro et al. with slight modifications [5, 43]. The animals were divided in two groups: control (CTL) and stressed (CVS); each group consisted of 10 animals. Rats in the control group were kept undisturbed in their home cages (5 rats in each cage of dimensions 65 × 25 cm, 18 cm high), while rats in the stressed group were exposed to various stress conditions for 40 days. In the experiment, the following stressors were used: 24 h of food deprivation, 24 h of water deprivation, 1–3 h of restraint, 1.5–2 h of restraint at 4°C, forced swimming for 10 or 15 min, flashing light for 120–210 min, and isolation (2-3 days). Specific stressors and length of time applied each day are listed in Table 1. To avoid predictability, rats were exposed to these stressors at different times each day.

The rat was placed inside a 26 × 6 cm plastic tube in order to restrain it, followed by plaster tape adjustments on the outside to prevent it from moving. Breathing was enabled through a 1 cm hole at the far end of the tube. Forced swimming was performed by placing the rat in a round glass tank (50 cm radius) filled with 23°C water. In order to expose the animal to the flashing light, we put the rat inside a 50 cm-high open-field container (40 × 60 cm) made of brown plywood with a frontal glass wall. A flashing light was delivered from a 40 W lamp, set at 60 flashes/min.

After 40 days of stress procedures and 24 h after the last stressor, the animals were decapitated and individual brain samples were washed with 20 mL of saline and stored at −75°C until the time of analysis.

### 2.3. Determination of Biochemical Parameters

Homogenates of the prefrontal cortex were prepared from frozen brain samples, using extraction buffer. All biochemical measurements were conducted from these homogenates. The experimental procedures were performed according to the instructions supplied with each respective kit. In this experiment, the following analysis was performed: lipid peroxidation (LPO), which was based on malondialdehyde and 4-hydroxyalkenals concentration (MDA + 4HAE) (OxisResearch, USA) and concentration of glutathione—GSH, GSSG, GSH/GSSG ratio (Calbiochem, Germany), glutathione peroxidase (GPX), and glutathione reductase (GR) activities (Cayman Chemical, USA). In short, the principle underlying lipid peroxidation assessment was based on the reaction of a chromogenic reagent R1 (N-methyl-2-phenylindole) with malondialdehyde (MDA) and 4-hydroxyalkenals (4HAE) at 45°C. Two molecules of R1 react with one molecule of MDA or 4-hydroxyalkenals to form a chromophore with an absorbance maximum at 586 nm. Measuring the concentration of MDA in combination with 4-hydroxyalkenals in methane sulfonic acid was used as an indicator of lipid peroxidation. GSH and GSSG concentrations were determined by an enzymatic reaction using Ellman's reagent (5,5′-dithiobis-2-nitrobenzoic acid) and reagent M2VP (1-methyl-2-vinyl-pyridine-trifluoro-methanesulfonamide sulfonate). In this method, GSH reacts with Ellman's reagent to form a product identified spectrophotometrically at a wavelength of *λ* = 412 nm. Oxidized glutathione (GSSG), produced upon reduction of an organic hydroperoxide by GPX, is recycled to its reduced state by GR and NADPH. The oxidation of NADPH to NADP^+^ is accompanied by a decrease in absorbance at 340 nm. GPX activity was measured by the kinetic method, using the aforementioned kit. In this method, GPX catalyzes the oxidation of glutathione by cumene hydroperoxide, and in the presence of GR and NADPH, the oxidized glutathione (GSSG) is immediately converted to a reduced form with a concomitant oxidation of NADPH to NADP^+^. The GR activity assay is based on the reduction of GSSG catalyzed by GR in the presence of NADPH, which is oxidized to NADP^+^. The reduction in absorbance was measured at 340 nm.

### 2.4. Determination of DNA Oxidative Damage

DNA was isolated with a Syngen DNA Mini Kit (Syngen, Poland) according to the manufacturer's protocol. The concentration and purity of the genomic DNA were measured using a NanoDrop MaestroNano Micro-Volume Spectrophotometer (Maestrogen Inc., Taiwan) and adjusted to 100 *μ*g/mL in TE buffer. Oxidative DNA damage was evaluated by measuring the amount of basic sites (the so-called AP) with a DNA Damage Quantification Kit (Dojindo, Japan). Oxidative attacks by ROS on the deoxyribose moiety lead to the release of free bases from DNA, generating strand breaks with various sugar modifications and simple abasic sites (AP sites). Aldehyde-reactive probe (ARP; N′-aminooxymethylcarbonylhydrazin-D-biotin) reacts specifically with an aldehyde group present on the open ring form of AP sites, making it possible to detect DNA modifications that result in the formation of an aldehyde group. Biotin-avidin-specific connection and horseradish peroxidase were used for colorimetric detection at 650 nm. AP sites were measured in DNA isolated from the hippocampus of the rats.

### 2.5. mRNA Expression Analysis

The isolated hippocampus was rinsed with 20 *μ*L of the solution used for injections and stored at −75°C until isolation of RNA was carried out. A quantitative real-time PCR (qPCR) method was used to evaluate expression of selected genes (*Ogg1*, *MsrA*, and *Nrf2*). RNA was isolated from 30 mg of tissue according to the manufacturer's instructions using Syngen Tissue RNA Mini Kit (Syngen Biotech, Poland). Reverse transcription was performed using NG dART RT-PCR kit (EURx, Poland) according to the manufacturer's instructions. The relative expression of genes was measured with the ΔΔCt method, using Hprt (Mn00446968_m1) as an endogenous control. The reaction was carried out in octuplicate by qPCR using the SmartChip Real-Time PCR System (WaferGen Bio-systems) and TaqMan Fast Universal PCR Master Mix (2x) (Applied BioSystems, USA) according to manufacturer's instructions. Sample quality screening based on amplification, Tm, and Ct values was performed to remove any outlier data points before ΔΔCt calculation and to determine fold change in mRNA levels. The data were presented as RQ value (RQ = 2 − ΔΔCt).

### 2.6. Statistical Analysis

The results were analysed statistically in the STATISTICA versus 10 application (StaftSoft, Cracow, Poland). Data was calculated as mean ± SEM and expressed as percentage of control group. The statistical significance among the groups was determined by a Student's *t*-test. All parameters were considered statistically significantly different if *p* values were less than 0.05.

## 3. Results

### 3.1. Lipid Peroxidation

**Figure 1:**
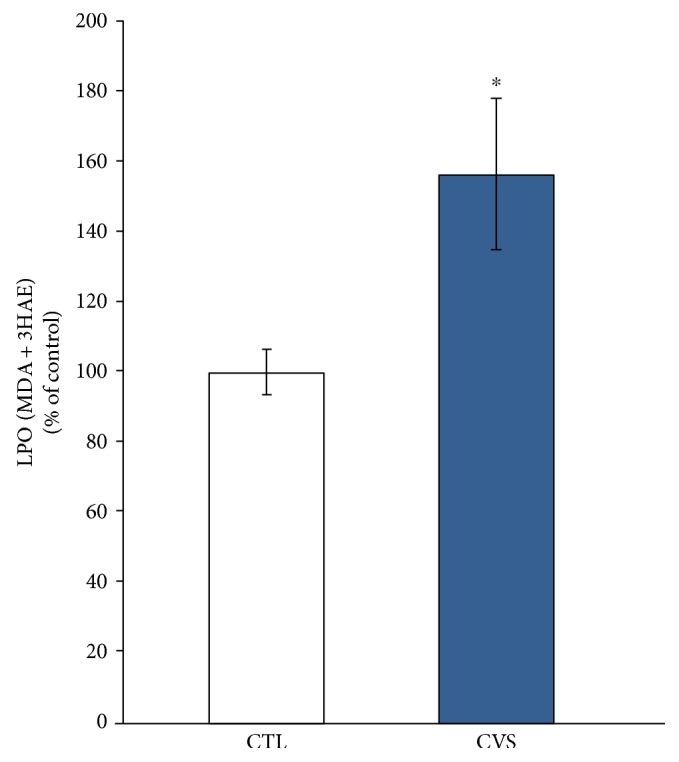
The effect of chronic variable stress on LPO (MDA + 4 HAE concentration) in the prefrontal cortex of rats. The method is based on the measurement of the following products of lipid peroxidation: malondialdehyde (MDA) and 4-hydroxyalkenals (4HAE), which react with a chromogenic reagent N-methyl-2-phenylindole. Data is displayed as mean ± SEM and expressed as percentage of control group. Significance: ^∗^*p* < 0.05 by Student's *t*-test; *p* = 0.0222; *t* = 2.503 with 18 degrees of freedom.

In this experiment, we evaluated lipid peroxidation by measuring malondialdehyde (MDA) and 4-hydroxyalkenals (4HAE) concentration in the prefrontal cortex of rats. MDA + 4HAE levels were significantly higher in the group of rats exposed to stress in comparison to the control group (Figure 1).

### 3.2. GSH and GSSG Levels

**Figure 2:**
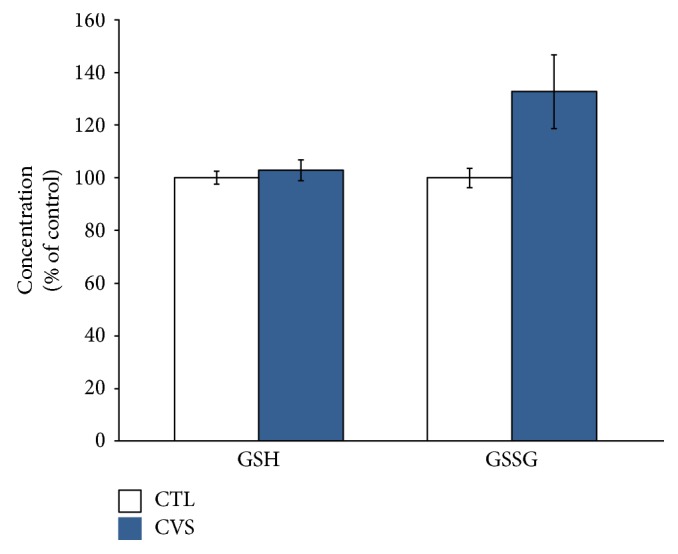
The effect of chronic variable stress on GSH and GSSG concentrations in the prefrontal cortex of rats. The concentrations were determined spectrophotometrically in an enzymatic reaction using the following reagents: 5,5′-dithiobis-2-nitrobenzoic acid and 1-methyl-2-vinyl-pyridine-trifluoro-methanesulfonamide sulfonate. Data is displayed as mean ± SEM and expressed as percentage of control group.

**Figure 3:**
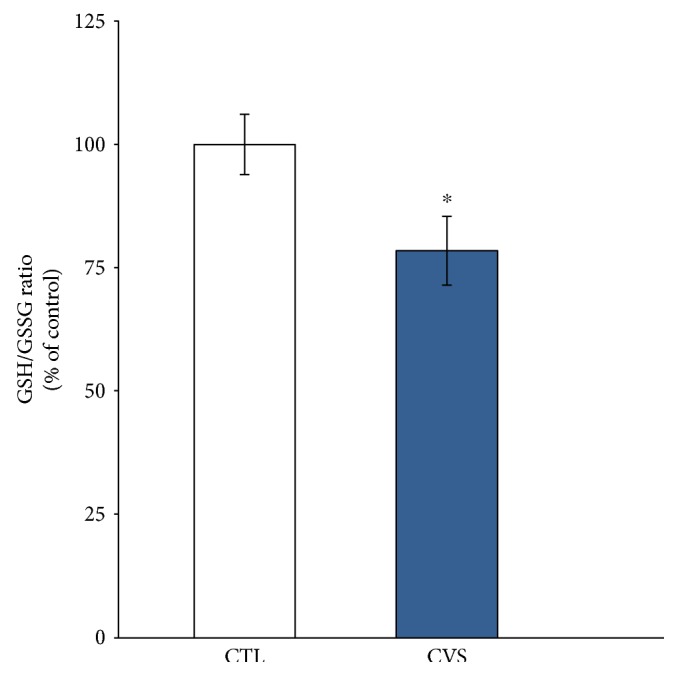
The effect of chronic variable stress on the GSH/GSSH ratio in the prefrontal cortex of rats. Data is displayed as mean ± SEM and expressed as percentage of control group. Significance: ^∗^*p* < 0.05 by Student's *t*-test; *p* = 0.0489; *t* = 2.320 with 8 degrees of freedom.

We noticed that the levels of GSH and GSSG in rats exposed to CVS did not change substantially. However, we also evaluated the GSH/GSSG ratio, since it is considered to be a sensitive indicator of the cellular redox state. Data obtained from the prefrontal cortex of rats submitted to chronic stress did show a statistically significant decrease in the GSH/GSSG ratio (Figures 2 and 3).

### 3.3. Glutathione Peroxidase and Glutathione Reductase Activity

**Figure 4:**
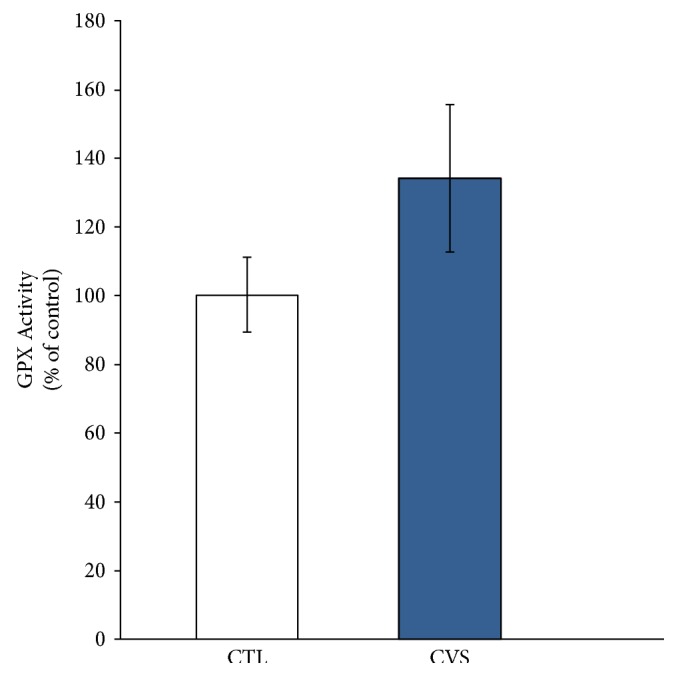
The effect of chronic variable stress on GPX activity in the prefrontal cortex of rats. The GPX activity was measured by a kinetic method based on the oxidation of NADPH to NADP^+^ which is accompanied by a decrease in absorbance at 340 nm. Data is displayed as mean ± SEM and expressed as percentage of control group.

**Figure 5:**
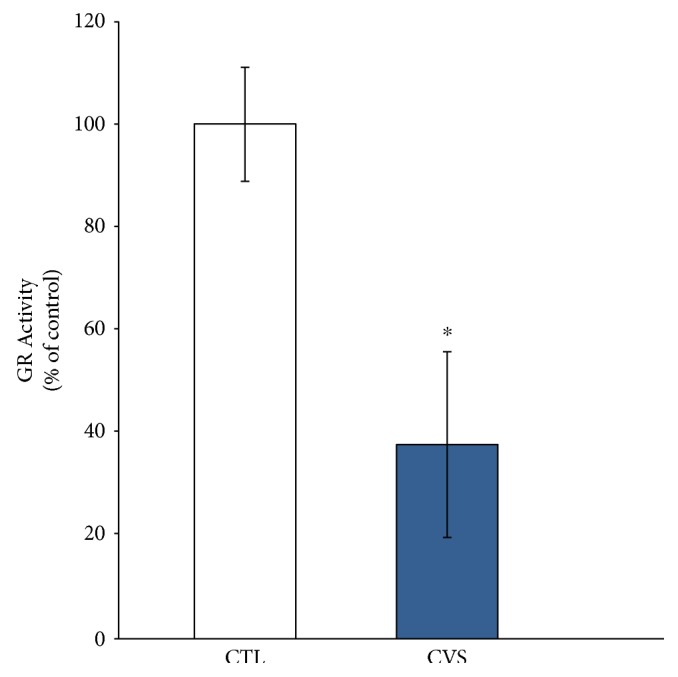
The effect of chronic variable stress on GR activity in the prefrontal cortex of rats. The GR activity was measured by kinetic method based on the oxidation of NADPH to NADP^+^ which is accompanied by a decrease in absorbance at 340 nm. Data is displayed as mean ± SEM and expressed as percentage of control group. Significance: ^∗^*p* < 0.05 by Student's *t*-test; *p* = 0.0189; *t* = 2.932 with 8 degrees of freedom.

No statistically significant changes were observed in glutathione peroxidase activity in rats subjected to CVS in comparison to the control group (Figure 4). However, the results indicate that stressors caused a decrease in glutathione reductase activity in the brain of rats exposed to CVS compared to the brain of control rats (Figure 5).

### 3.4. Oxidative DNA Damage

**Figure 6:**
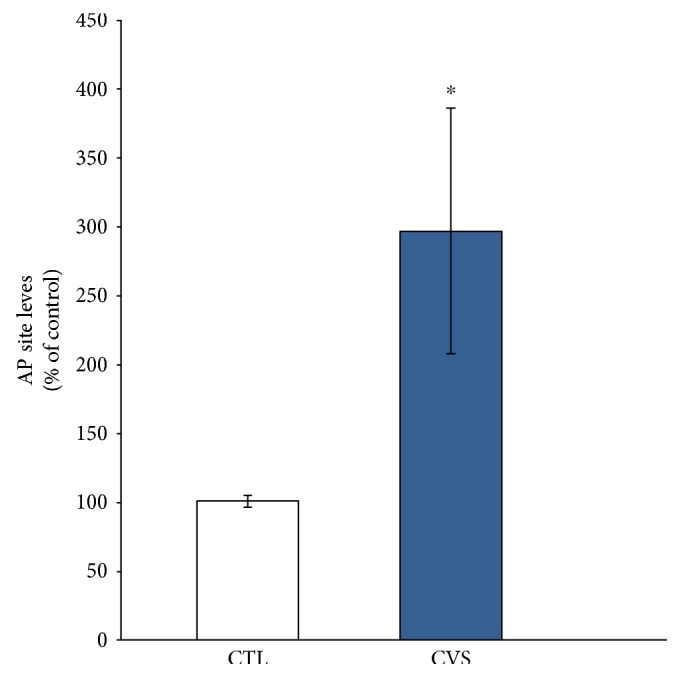
The effect of chronic variable stress on oxidative damages of DNA in the hippocampus of rats. The method is based on the measurement of simple abasic sites (AP sites) in DNA. Data is displayed as mean ± SEM and expressed as percentage of control group. Significance: ^∗^*p* < 0.05 by Student's *t*-test; *p* = 0.0391; *t* = 2.194 with 22 degrees of freedom.

Assessment of oxidative DNA damage showed a threefold increase of AP site accumulation in DNA isolated from the hippocampus of rats subjected to CVS in comparison to the control group. This result demonstrates that the amount of oxidative damage in stressed rats rose significantly compared to controls (Figure 6).

### 3.5. The Level of mRNA Expression for *Ogg1*, *MsrA*, and *Nrf2*

**Figure 7:**
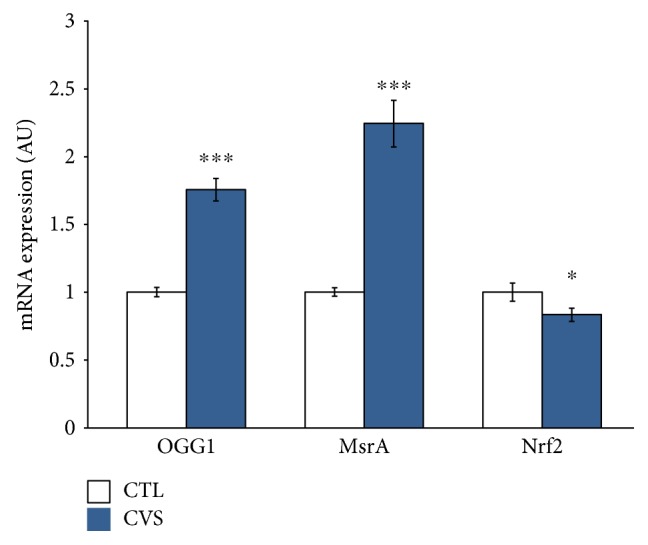
The effect of chronic variable stress on the level of mRNA expression for OGG1, MsrA, and Nrf2 genes in the hippocampus of rats. The mRNA's expression was assayed in regard to the control group, where the expression level is estimated at 1. Data is displayed as mean ± SEM. Significance: ^∗^*p* < 0.05; ^∗∗∗^*p* < 0.001 by Student's *t*-test; OGG1: *p* < 0.0001 and *t* = 8.408 with 18 degrees of freedom; MsrA: *p* < 0.0001 and *t* = 7.155 with 18 degrees of freedom; Nrf2: *p* = 0.0422 and *t* = 2.091 with 45 degrees of freedom.

The expression levels of genes involved in oxidative stress were examined. Sample variation was accounted for by comparison to the expression levels of Hprt, which is a housekeeping gene responsible for nucleotide metabolism. Expression of mRNA was measured in reference to the control group, where the expression level is estimated as RQ = 1. In the hippocampus of rats submitted to CVS, the mRNA levels of *Ogg1* and *MsrA* were significantly increased, while a decrease in the expression of the *Nrf2* gene was noticed in comparison to the control. The results of qRT-PCR experiments are shown in Figure 7.

## 4. Discussion

It is postulated that environmental stress, at least partially, is related to oxidative stress. The main source of ROS formation is mitochondrial failure, that is, associated with neurodegeneration [48]. In stressogenic-related depressive disorders, the rate of conversion of the oxygen to ROS may increase and can result in severe metabolic dysfunction and oxidative damages to subcellular and cellular membrane lipids and enzymes [8, 12–14]. However, one of the most important consequences of ROS overproduction is modification of DNA. For these reasons, the objective of our study was to evaluate oxidative DNA damage in the brain of rats submitted to chronic variable stress. We also measured the biochemical changes associated with ROS generation, which can confirm the occurrence of oxidative stress.

The current study revealed an increase in lipid peroxidation in the prefrontal cortex of rats exposed to stress, which indicates oxidative stress. It is coherent with the results of the clinical tests, which indicate that the pathological stress causing a huge depression is accompanied by an increase of lipid peroxidation in the brain [8, 11, 17]. Highly reactive oxygen metabolites act on unsaturated fatty acids of phospholipid components of membranes to produce malondialdehyde, a lipid peroxidation product. Reactivity of MDA and 4-HNE may cause damage to DNA [20]. In the brain, there is a significant amount of unsaturated fatty acids, which are susceptible to peroxidation. Moreover, in the brain, there is a relatively poor antioxidant defense system. This creates a risk of DNA damage and disturbances in secondary electron transport and cellular damage [49]. Lipid peroxidation decreases the life span of neurons, affects neurotransmitter release, and was reported as a major contributor to the loss of cell function under oxidative stress conditions in depression [26, 50].

In our study, exposure to stress caused a decrease of the GSH/GSSG ratio in the prefrontal cortex of rats in comparison to control. Glutathione plays an important role in a multitude of cellular processes, including cell differentiation, proliferation, and apoptosis. GSH is critical for protecting the brain from oxidative stress, acting as a free radical scavenger and inhibitor of lipid peroxidation. The GSH/GSSG ratio is reduced in neurodegenerative diseases [51]. The brain is particularly susceptible to alterations in GSH homeostasis, probably due to the fact that GSH may be a neuromodulator or neurotransmitter and may thus be essential for central nervous system activities [48]. The oxidation of glutathione is considered to be one of the first and most important events leading to a change in the overall cellular redox state. The resulting damage is thought to be involved in neurodegenerative diseases [48, 52]. Thus, the GSH/GSSG ratio is considered to be a sensitive indicator of the cellular redox state [53, 54]. Taking this into account, the increase in LPO accompanied by the decrease in the GSH/GSSG ratio may suggest that the antioxidative adaptation is not sufficient. A decrease in the GSH/GSSG ratio manifests itself largely through an increased susceptibility to oxidative stress, and the resulting damage is thought to be involved in neurodegenerative diseases [48].

In addition to the GSH/GSSG ratio, the relative activities of the enzymes responsible for glutathione metabolism are an important factor for assessing the redox potential of tissues and cells. For this reason, glutathione peroxidase and glutathione reductase activities were measured. They are enzymatic antioxidant system chains and provide protection against the damaging effects of free radicals [52, 55]. In this system, glutathione peroxidase provides detoxification of organic and inorganic peroxides by using reduced glutathione. Glutathione reductase regenerates GSH and protects cell from death caused by oxidative stress, probably through maintaining a high GSH/GSSG ratio [54, 56]. This experiment showed that GR activity is decreased in the prefrontal cortex of stressed rats. The decrease in GR activity, observed in our work, may result in impairment of GSH regeneration. This statement is confirmed by the decrease in the GSH/GSSG ratio. Perhaps, regulation of GR is associated with cortisol, which is secreted in response to stress. Becerril-Chavez et al. recently showed that the activity levels of glutathione-related enzymes (GPX, GST) are disrupted in the prefrontal cortex of rats subjected to chronic stress. Their research proved that downregulation of glutathione S-transferase (GST) occurred [57]. This enzyme catalyzes deactivation of many harmful substances and requires reduced glutathione as a cofactor. In turn, glutathione reductase is responsible for the restoration of GSH. The decrease in GR activity may result in insufficient GSH regeneration and can adversely affect GST activity. The GST dysfunction was accompanied by a high cortisol level, which increases in response to an acutely stressful event [57]. Hence, chronic stress mounts a stronger corresponding cortisol response [9]. Importantly, the elevated cortisol level was decreased by application of an antioxidant, which also attenuated the GST activity level. However, further studies are required to confirm these correlations. So far, the above results are indirect evidence of ROS overproduction and presence of disrupted oxidative defense systems.

In stress-induced depressive disorders affecting the brain, DNA damage induced by oxidative stress is a major factor leading to neuronal dysfunction and cell death. Studies have shown a relationship between DNA damage and cortisol, especially during prolonged exposure to high levels of cortisol, in brain structures such as the hippocampus and frontal cortex [10]. Oxidant-induced DNA damage may be a useful biomarker for chronic oxidative stress determination [58]. Oxidative damage of DNA results from an interaction of DNA with reactive oxygen species, in particular the hydroxyl radical. Oxidative damage is believed to contribute substantially to the decline in cellular functions that are associated with nervous system diseases [59]. Moreover, studies have shown that oxidative DNA damage is linked to the onset of specific human diseases such as neuronal degeneration [58–60]. One of the major types of damage generated by ROS is the AP site, the most common DNA damage resulting from loss of a DNA base [61]. The current study revealed a statistically significant increase of AP site accumulation in DNA isolated from the hippocampus of rats subjected to CVS compared to that of the control group, an indicator of considerable oxidative DNA damage. An important role in the repair of oxidatively damaged proteins is to restore biological activity through specific gene activation. The *Ogg1* gene is induced by oxidative stress and its mRNA levels are correlated with base excision capacity. OGG1 is ubiquitously expressed in the brain and is considered a cellular marker for both oxidative stress and oxidative DNA damage [23]. Protein-bound methionine residues are the most susceptible to oxidation by ROS. However, this modification can be repaired by *MsrA*, which catalyzes the thioredoxin-dependent reduction of free and protein-bound MetO to methionine [31].

The current study revealed that, in the hippocampus of rats subjected to CVS, the mRNA levels of *Ogg1* and *MsrA* were significantly increased. Taking into consideration the results of related biochemical research, we can assume that overexpression of DNA repair enzymes, important for maintenance of mitochondrial functions, may result out of necessity to rescue cells from ROS. Disruption of antioxidant and DNA repair mechanisms in the cell by ROS may result in oxidative stress and oxidative damage to the cell. Moskovitz et al. showed that loss of antioxidant capacity is associated with a decrease in total OGG1 and MSRA activities [31]; thus, the significant increase in expression of these genes shown in our study suggests that they play an important role in the protection against ROS-related oxidative DNA damage. The results presented above confirm that chronic environmental stress-induced oxidative stress causes DNA oxidative damage. At the same time, upregulation of *Ogg1* and *MsrA* increases efficiency of DNA repair.

Studies have determined that NRF2 is a regulator of antioxidant response and is a factor that regulates the transcription of oxidative DNA damage repair genes [62]. Oxidative stress can lead to NRF2 activation, which in turn acts as an autoregulatory feed-forward loop to dampen the increased ROS levels, thereby maintaining homeostasis following tissue or cellular injury. NRF2 activation would be expected in the presence of an increase in ROS. However, in our study, a decrease in *Nrf2* gene expression was observed in the brain of rats submitted to chronic stress in comparison to the control group. One explanation for these findings may be that NRF2 is not properly responding to oxidative stress under conditions of chronic stress. Disturbed NRF2 signaling in the brain may contribute to neurodegeneration via decreased antioxidative defense as documented in depressed patients [32, 63]. This phenomenon has also been confirmed by studies that reported decreased *Nrf2* expression in the hippocampus of rats submitted to chronic stressors [64, 65]. The increase in *Ogg1* and *MsrA* expression observed in our work seems to be independent from *Nrf2* expression levels. The obtained data suggest that oxidative stress directly activates the oxidative stress response genes but not the NRF2 pathway. These results support the theory that oxidative stress responses do not always involve a coordinated regulation of genes and their activities are regulated by different factors. The second explanation for the decrease in activity of Nrf2 gene is its exceptional sensitivity for oxidative products (ROS) and byproducts (MDA and 4-HNE), but this assumption should be supported by future studies.

The redox-sensitive transcription factor NRF2 also regulates the rate of GSH synthesis [66]. GSH-metabolizing enzymes are induced at the transcriptional level by mild oxidative stress, which involves binding of the NRF2 transcription factor to the antioxidant response element [67]. Taking into account the above results, downregulation of *Nrf2* may be correlated with decreased GR activity and, consequently, could lead to a decrease in the GSH/GSSG ratio, as recorded in our study. The decrease of *Nrf2* is corresponding with the data from biochemical studies and points on disorders in defense of oxidative system.

## 5. Conclusions

The current study confirmed that chronic variable stress causes oxidative stress in part of the brain involved in depression development, that is, the prefrontal cortex and hippocampus. In the hippocampus, an increase in oxidative damages of DNA was noticed. Our finding of *Ogg1* and *MsrA* upregulation indicates that the oxidative DNA repair system has been activated. The decrease of *Nrf2* may suggest an independent activation of oxidative stress response genes. Future studies are required to explain if oxidative damage of DNA originates from mitochondria or nucleus and if the decrease in important antioxidative *Nrf2* gene activity is related to special susceptibility for oxidative stress byproducts. This may help in clarifying whether oxidative stress is the cause or a downstream consequence of depression.

## Figures and Tables

**Table 1:** Stressor agents used during the chronic variable stress.

Day of treatment	Stressor	Duration	Start time
1	Water deprivation	24 h	8:00 a.m.
2	Food deprivation	24 h	9:00 a.m.
3	Isolation	24 h	10:00 a.m.
4	Isolation	24 h	10:00 a.m.
5	Isolation	24 h	10:00 a.m.
6	Flashing light	3 h	12:00 a.m.
7	Food deprivation	24 h	8:00 a.m.
8	Forced swimming	10 min	9:00 a.m.
9	Restraint	1 h	11:00 a.m.
10	Water deprivation	24 h	9:00 a.m.
11	No stressor	—	—
12	No stressor	—	—
13	Restraint and cold	2 h	10:00 a.m.
14	Flashing light	2.5 h	9:00 a.m.
15	Food deprivation	24 h	8:00 a.m.
16	Forced swimming	15 min	12:00 a.m.
17	Isolation	24 h	8:00 a.m.
18	Isolation	24 h	8:00 a.m.
19	Isolation	24 h	8:00 a.m.
20	Water deprivation	24 h	10:00 a.m.
21	Food deprivation	24 h	9:00 a.m.
22	Flashing light	3 h	13:00 a.m.
23	Restraint	2 h	12:00 a.m.
24	Isolation	24 h	8:00 a.m.
25	Isolation	24 h	8:00 a.m.
26	Restraint and cold	1.5 h	12:00 a.m.
27	Forced swimming	10 min	10:00 a.m.
28	Flashing light	3.5 h	12:00 a.m.
29	No stressor	—	—
30	Food deprivation	24 h	8:00 a.m.
31	Restraint	3 h	9:00 a.m.
32	Flashing light	2 h	10:00 a.m.
33	Water deprivation	24 h	8:00 a.m.
34	Restraint and cold	2 h	10:00 a.m.
35	Forced swimming	15 min	11:00 a.m.
36	Isolation	24 h	8:00 a.m.
37	Isolation	24 h	8:00 a.m.
38	No stressor	—	—
39	Flashing light	3 h	13:00 a.m.
40	Forced swimming	10 min	8:00 a.m.

## Additional Points

*Compliance with ethical standards*. All procedures were conducted in accordance with the European Communities Council Directive of September 22, 2010 (2010/63/EU), and Polish legislation acts concerning animal experimentations. The experimental procedures and protocols were approved by the First Local Ethics Committee at the Medical University of Lublin.

## References

[1] Hashimoto K. (2011). The role of glutamate on the action of antidepressants. *Progress in Neuro-Psychopharmacology & Biological Psychiatry*.

[2] Kessler R. C., Berglund P., Demler O. (2003). The epidemiology of major depressive disorder: results from the National Comorbidity Survey Replication (NCS-R). *JAMA*.

[3] Du J., Zhu M., Bao H. (2016). The role of nutrients in protecting mitochondrial function and neurotransmitter signaling: implications for the treatment of depression, PTSD, and suicidal behaviors. *Critical Reviews in Food Science and Nutrition*.

[4] Robinson M. E., Teyhen D. S., Wu S. S. (2009). Mental health symptoms in combat medic training: a longitudinal examination. *Military Medicine*.

[5] Tagliari B., Noschang C. G., Ferreira A. G. (2010). Chronic variable stress impairs energy metabolism in prefrontal cortex and hippocampus of rats: prevention by chronic antioxidant treatment. *Metabolic Brain Disease*.

[6] Fontella F. U., Siqueira R. I., Vasconcellos A. P. S., Tabajara A. S., Netto C. A., Dalmaz C. (2005). Repeated restraint stress induces oxidative damage in rat hippocampus. *Neurochemical Research*.

[7] Manoli L. P., Gamaro G. D., Silveira P. P., Dalmaz C. (2000). Effect of chronic variate stress on thiobarbituric-acid reactive species and on total radical-trapping potential in distinct regions of rat brain. *Neurochemical Research*.

[8] Blici M., Efe H., Köroğlu M. A., Uydu H. A., Bekaroğlu M., Değer O. (2001). Antioxidative enzyme activities and lipid peroxidation in major depression: alterations by antidepressant treatments. *Journal of Affective Disorders*.

[9] Aschbacher K., O'Donovan A., Wolkowitz O. M., Dhabhar F. S., Su Y., Epel E. (2013). Good stress, bad stress and oxidative stress: insights from anticipatory cortisol reactivity. *Psychoneuroendocrinology*.

[10] Şimşek Ş., Yüksel T., Kaplan İ., Uysal C., Aktaş H. (2016). The levels of cortisol and oxidative stress and DNA damage in child and adolescent victims of sexual abuse with or without post-traumatic stress disorder. *Psychiatry Investigation*.

[11] Michel T. M., Pulschen D., Thome J. (2012). The role of oxidative stress in depressive disorders. *Current Pharmaceutical Design*.

[12] Roediger B., Armati P. J. (2003). Oxidative stress induces axonal beading in cultured human brain tissue. *Neurobiology of Disease*.

[13] Lucca G., Comim C. M., Valvassori S. S. (2009). Increased oxidative stress in submitochondrial particles into the brain of rats submitted to the chronic mild stress paradigm. *Journal of Psychiatric Research*.

[14] Valavanidis A., Vlachogianni T., Fiotakis K., Loridas S. (2013). Pulmonary oxidative stress, inflammation and cancer: respirable particulate matter, fibrous dusts and ozone as major causes of lung carcinogenesis through reactive oxygen species mechanisms. *International Journal of Environmental Research and Public Health*.

[15] Torres R. L., Torres I. L. S., Gamaro G. D. (2004). Lipid peroxidation and total radical-trapping potential of the lungs of rats submitted to chronic and sub-chronic stress. *Brazilian Journal of Medical and Biological Research*.

[16] Brenner-Lavie H., Klein E., Ben-Shachar D. (2009). Mitochondrial complex I as a novel target for intraneuronal DA: modulation of respiration in intact cells. *Biochemical Pharmacology*.

[17] Bajpai A., Verma A. K., Srivastava M., Srivastava R. (2014). Oxidative stress and major depression. *Journal of Clinical and Diagnostic Research*.

[18] Hausladen A., Fridovich I. (1994). Superoxide and peroxynitrite inactivate aconitases, but nitric oxide does not. *The Journal of Biological Chemistry*.

[19] Lemasters J. J., Nieminen A. L. (2001). *Mitochondria in Pathogenesis*.

[20] Ayala A., Muñoz M. F., Argüelles S. (2014). Lipid peroxidation: production, metabolism, and signaling mechanisms of malondialdehyde and 4-hydroxy-2-nonenal. *Oxidative Medicine and Cellular Longevity*.

[21] Popa-Wagner A., Mitran S., Sivanesan S., Chang E., Buga A. M. (2013). ROS and brain diseases: the good, the bad, and the ugly. *Oxidative Medicine and Cellular Longevity*.

[22] Zhou F., Zhang W., Wei Y. (2007). The changes of oxidative stress and human 8-hydroxyguanine glycosylase1 gene expression in depressive patients with acute leukemia. *Leukemia Research Reports*.

[23] Teyssier J. R., Ragot S., Chauvet-Gélinier J. C., Trojak B., Bonin B. (2011). Expression of oxidative stress-response genes is not activated in the prefrontal cortex of patients with depressive disorder. *Psychiatry Research*.

[24] Davies K. J. A. (2000). Oxidative stress, antioxidant defenses, and damage removal, repair, and replacement systems. *IUBMB Life*.

[25] Powell C. L., Swenberg J. A., Rusyn I. (2005). Expression of base excision DNA repair genes as a biomarker of oxidative DNA damage. *Cancer Letters*.

[26] Vaváková M., Ďuračková Z., Trebatická J. (2015). Markers of oxidative stress and neuroprogression in depression disorder. *Oxidative Medicine and Cellular Longevity*.

[27] Fukae J., Takanashi M., Kubo S. (2005). Expression of 8-oxoguanine DNA glycosylase (OGG1) in Parkinson’s disease and related neurodegenerative disorders. *Acta Neuropathologica*.

[28] Dizdarouglu M. (1991). Chemical determination of free radical-induced damage to DNA. *Free Radical Biology and Medicine*.

[29] Maes M., Mihaylova I., Kubera M., Uytterhoeven M., Vrydags N., Bosmans E. (2009). Increased 8-hydroxy-deoxyguanosine, a marker of oxidative damage to DNA, in major depression and myalgic encephalomyelitis/chronic fatigue syndrome. *Neuroendocrinology Letters*.

[30] Moskovitz J., Flescher E., Berlett B. S., Azare J., Poston J. M., Stadtman E. R. (1998). Overexpression of peptide-methionine sulfoxide reductase in *Saccharomyces cerevisiae* and human T cells provides them with high resistance to oxidative stress. *Proceedings of the National Academy of Sciences of the United States of America*.

[31] Moskovitz J., Bar-Noy S., Williams W. M., Requena J., Berlett B. S., Stadtman E. R. (2001). Methionine sulfoxide reductase (MsrA) is a regulator of antioxidant defense and lifespan in mammals. *Proceedings of the National Academy of Sciences of the United States of America*.

[32] de Vries H. E., Witte M., Hondius D. (2008). Nrf2-induced antioxidant protection: a promising target to counteract ROS-mediated damage in neurodegenerative disease?. *Free Radical Biology and Medicine*.

[33] Nguyen T., Nioi P., Pickett C. B. (2009). The Nrf2-antioxidant response element signaling pathway and its activation by oxidative stress. *The Journal of Biological Chemistry*.

[34] Martín-de-Saavedra M. D., Budni J., Cunha M. P. (2013). Nrf2 participates in depressive disorders through an anti-inflammatory mechanism. *Psychoneuroendocrinology*.

[35] Mendez-David I., Tritschler L., Ali Z. E. (2015). Nrf2-signaling and BDNF: a new target for the antidepressant-like activity of chronic fluoxetine treatment in a mouse model of anxiety/depression. *Neuroscience Letters*.

[36] Ma Q. (2013). Role of Nrf2 in oxidative stress and toxicity. *Annual Review of Pharmacology and Toxicology*.

[37] Kovac S., Angelova P. R., Holmström K. M., Zhang Y., Dinkova-Kostova A. T., Abramov A. Y. (2015). Nrf2 regulates ROS production by mitochondria and NADPH oxidase. *Biochimica et Biophysica Acta*.

[38] Kobayashi M., Yamamoto M. (2005). Molecular mechanisms activating the Nrf2-Keap1 pathway of antioxidant gene regulation. *Antioxidants and Redox Signaling*.

[39] Tomobe K., Shinozuka T., Kuroiwa M., Nomura Y. (2012). Age-related changes of Nrf2 and phosphorylated GSK-3β in a mouse model of accelerated aging (SAMP8). *Archives of Gerontology and Geriatrics*.

[40] Lorenzetti V., Allen N. B., Fornito A., Yücel M. (2009). Structural brain abnormalities in major depressive disorder: a selective review of recent MRI studies. *Journal of Affective Disorders*.

[41] Gawryluk J. W., Wang J. F., Andreazza A. C., Shao L., Young L. T. (2011). Decreased levels of glutathione, the major brain antioxidant, in post-mortem prefrontal cortex from patients with psychiatric disorders. *International Journal of Neuropsychopharmacology*.

[42] Andrus B. M., Blizinsky K., Vedell P. T. (2012). Gene expression patterns in the hippocampus and amygdala of endogenous depression and chronic stress models. *Molecular Psychiatry*.

[43] Gamaro G. D., Manoli L. P., Torres I. L. S., Silveira R., Dalmaz C. (2003). Effects of chronic variate stress on feeding behavior and on monoamine levels in different rat brain structures. *Neurochemistry International*.

[44] Tagliari B., dos Santos T. M., Cunha A. A. (2010). Chronic variable stress induces oxidative stress and decreases butyrylcholinesterase activity in blood of rats. *Journal of Neural Transmission*.

[45] Zhao Z., Wang W., Guo H., Zhou D. (2008). Antidepressant-like effect of liquiritin from *Glycyrrhiza uralensis* in chronic variable stress induced depression model rats. *Behavioral Brain Research*.

[46] Krishnan V., Nestler E. J. (2011). Animal models of depression: molecular perspectives. *Current Topics in Behavioral Neurosciences*.

[47] Raudkivi K., Mällo T., Harro J. (2012). Effect of chronic variable stress on corticosterone levels and hippocampal extracellular 5-HT in rats with persistent differences in positive affectivity. *Acta Neuropsychiatrica*.

[48] Ballatori N., Krance S. M., Notenboom S., Shi S., Tieu K., Hammond C. L. (2009). Glutathione dysregulation and the etiology and progression of human diseases. *Biological Chemistry*.

[49] Ott M., Gogvadze V., Orrenius S., Zhivotovsky B. (2007). Mitochondria, oxidative stress and cell death. *Apoptosis*.

[50] Storey K. B. (1996). Oxidative stress: animal adaptations in nature. *Brazilian Journal of Medical and Biological Research*.

[51] Owen J. B., Butterfield D. A. (2010). Measurement of oxidized/reduced glutathione ratio. *Methods in Molecular Biology*.

[52] Kaushik S., Kaur J. (2003). Chronic cold exposure affects the antioxidant defense system in various rat tissues. *Clinica Chimica Acta*.

[53] Faria R., Santana M. M., Aveleira C. A. (2014). Alterations in phospholipidomic profile in the brain of mouse model of depression induced by chronic unpredictable stress. *Neuroscience*.

[54] Jones D. P. (2002). Redox potential of GSH/GSSG couple: assay and biological significance. *Methods in Enzymology*.

[55] Paglia D. E., Valentine W. N. (1967). Studies on the quantitative and qualitative characterization of erythrocyte glutathione peroxidase. *Journal of Laboratory and Clinical Medicine*.

[56] Yang M. S., Chan H. W., Yu L. C. (2006). Glutathione peroxidase and glutathione reductase activities are partially responsible for determining the susceptibility of cells to oxidative stress. *Toxicology*.

[57] Becerril-Chavez H., Colin-Gonzalez A. L., Villeda-Hernandez J. (2017). Protective effects of S-allyl cysteine on behavioral, morphological and biochemical alterations in rats subjected to chronic restraint stress: antioxidant and anxiolytic effects. *Journal of Functional Foods*.

[58] Yakes F. M., Van Houten B. (1997). Mitochondrial DNA damage is more extensive and persists longer than nuclear DNA damage in human cells following oxidative stress. *Proceedings of the National Academy of Sciences of the United States of America*.

[59] Englander E. W. (2008). Brain capacity for repair of oxidatively damaged DNA and preservation of neuronal function. *Mechanisms of Ageing and Development*.

[60] Huang D., Shenoy A., Cui J., Huang W., Lui P. K. (2000). In situ detection of AP sites and DNA strand breaks bearing 3'-phosphate termini in ischemic mouse brain. *FASEB Journal*.

[61] Filler K., Lyon D., Bennett J. (2014). Association of mitochondrial dysfunction and fatigue: A review of the literature. *Biochimica et Biophysica Acta Clinical*.

[62] Singh B., Chatterjee A., Ronghe A. M., Bhat N. K., Bhat H. K. (2014). Antioxidant-mediated up-regulation of OGG1 via NRF2 induction is associated with inhibition of oxidative DNA damage in estrogen-induced breast cancer. *BioMed Central Cancer*.

[63] Maes M., Galecki P., Chang Y. S., Berk M. (2011). A review on the oxidative and nitrosative stress (O&NS) pathways in major depression and their possible contribution to the (neuro)degenerative processes in that illness. *Progress in Neuropsychopharmacology and Biological Psychiatry*.

[64] Djordjevic J., Djordjevic A., Adzic M., Radojcic M. B. (2010). Chronic social isolation compromises the activity of both glutathione peroxidase and catalase in hippocampus of male wistar rats. *Cellular and Molecular Neurobiology*.

[65] Djordjevic J., Djordjevic A., Adzic M., Mitic M., Lukic I., Radojcic M. B. (2015). Alterations in the Nrf2-Keap1 signaling pathway and its downstream target genes in rat brain under stress. *Brain Research*.

[66] Steele M. L., Fuller S., Patel M., Kersaitis C., Ooi L., Münch G. (2013). Effect of Nrf2 activators on release of glutathione, cysteinylglycine and homocysteine by human U373 astroglial cells. *Redox Biology*.

[67] Lushchak V. I. (2012). Glutathione homeostasis and functions: potential targets for medical interventions. *Journal of Amino Acids*.

